# Development of a smart spectral analysis method for the determination of mulberry (*Morus alba var. nigra* L.) juice quality parameters using FT‐IR spectroscopy

**DOI:** 10.1002/fsn3.3211

**Published:** 2022-12-27

**Authors:** Maryam Soltanikazemi, Saman Abdanan Mehdizadeh, Mokhtar Heydari, Seyed Mojtaba Faregh

**Affiliations:** ^1^ Department of Mechanics of Biosystems Engineering, Faculty of Agricultural and Rural Development Agricultural Sciences and Natural Resources University of Khuzestan Mollasani Iran; ^2^ Department of Horticulture, Faculty of Agriculture Agricultural Sciences and Natural Resources University of Khuzestan Mollasani Iran

**Keywords:** ANN, PLS, spectroscopy, ν‐SVR

## Abstract

Recently, the application of Fourier transform infrared (FT‐IR) spectroscopy as a noninvasive technique combined with chemometric methods has been widely noted for quality evaluation of agricultural products. Mulberry (*Morus alba var. nigra* L.) is a native fruit of Iran and there is limited information about its quality characteristics. The present study aims at assessing a nondestructive optical method for determining the internal quality of mulberry juice. To do so, first, FT‐IR spectra were acquired in the spectral range 1000–8333 nm. Then, the principal component analysis (PCA) was used to extract the principal components (PCs) which were given as inputs to three predictive models (support vector regression (SVR), partial least square (PLS), and artificial neural network (ANN)) to predict the internal parameters of the mulberry juice. The performance of predictive models showed that SVR got better results for the prediction of ascorbic acid (*R* ^2^ = .84, RMSE = 0.29), acidity (*R* ^2^ = .71, RMSE = 0.0004), phenol (*R* ^2^ = .35, RMSE = 0.19), total anthocyanin (*R* ^2^ = .93, RMSE = 5.85), and browning (*R* ^2^ = .89, RMSE = 0.062) compared to PLS and ANN. However, the ANN predicted the parameters TSS (*R* ^2^ = .98, RMSE = 0.003) and pH (*R* ^2^ = .99, RMSE = 0.0009) better than the other two models. The results indicated that a good prediction performance was obtained using the FT‐IR technique along with SVR and this method could be easily adapted to detect the quality parameters of mulberry juice.

## Graphical abstract

The aim of this work was to evaluate the potential of FT‐IR spectroscopy, as a quantitative analytical technique for the evaluation of TSS, ascorbic acid, acidity, phenol, anthocyanin, browning, and pH. In this study, PCA was used to extract features, and the ν‐SVR, PLS, and ANN were used as predictive models. Finally, the ability of each model to predict the internal parameters of mulberry juice is examined.

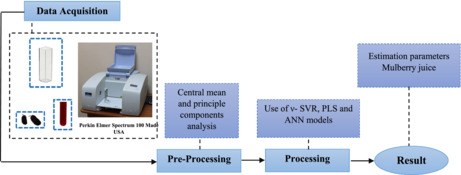

## 1 INTRODUCTION

Mulberry (*Morus alba var. nigra* L.) is a rich source of red pigments and anthocyanins (Zhang et al., 2018). In addition, the fruit has various medicinal properties, including antidiabetic properties (Asano et al., 2001; Shivangi et al., 2021), antiallergens (Andallu & Varadacharyulu, 2003), antiviral (Du et al., 2003), antioxidant (Hu et al., 2021), antidepressant (El‐Beshbishy et al., 2006), and neuroprotective (Kang et al., 2006). Today, due to the nutrient value of the mulberry fruits, they are eaten both fresh and in various other forms such as syrup, jam, pulp, ice cream, and vinegar (Gundogdu et al., 2011). Recent research has revealed that mulberry fruits produce positive effects on the human diet and health due to the help of its compounds such as the presence of fats and significant variation in fatty acids, ascorbic acid, minerals, phenols, and flavonoids (Ercisli & Orhan, 2007), some organic acids (Koyuncu, 2004,) and anthocyanin content (Lo Bianco & Mirabella, 2018; Przeor et al., 2020). However, the most important property of mulberry fruit for its pharmaceutical value is its antioxidant capacity (Wang et al., 2022). Özgen et al. (2009) stated that mulberry fruit had essential effects on human health because of its antioxidant contents, acidity, and sugar content.

Evaluation of internal quality attributes of agricultural products is one of the most important operations in postharvest management. The evaluation method for the assessment of nutritional value and fruit quality during the ripening process has to be simple, accurate, quick, and also nondestructive. One of these techniques is Fourier transform infrared (FTIR) spectroscopy, which, recently, has emerged as a nondestructive tool for agricultural applications and has made significant progress in the field of agricultural material evaluation. FTIR spectroscopy is relatively simple, reproducible, and nondestructive to the tissue, and only small amounts of material (micrograms to nanograms) with a minimum sample preparation are required. In addition, this technique also provides molecular‐level information allowing the investigation of functional groups, bonding types, and molecular conformations. Fourier transform infrared (FT‐IR) spectroscopy is often coupled with chemometrics and used to study different quality attributes in many food samples such as quantification of ascorbic acid in powdered mixture and liquid (Yang & Irudayaraj, 2002), measurements of internal quality of “Fuji” apples (Liu & Ying, 2005), and determination of polymethoxylated flavone in orange oil residues (Manthey, 2006).

Fourier transform infrared spectroscopy has been widely used for must and wine analysis (Thanasi et al., 2022; Topala & Tataru, 2019). Moreover, it has become an alternative method for sugar analysis (Masithoh et al., 2022) in food such as mango juices (Duarte et al., 2002), soft drinks, and fruit juices (Ramasami et al., 2004), rapid quality control of spirit drinks and beer (Lachenmeier, 2007), determination of crude protein and intestinal protein digestibility of wheat (Shi et al., 2019), predicting calcium in grape must and base wine (Véstia et al., 2019), and determination of main fruits in adulterated nectars (Miaw et al., 2018).

In spite of the emerging potential role of mulberry juice in benefiting good health, in the literature, the application of spectroscopy techniques for evaluating the quality parameters of mulberry juice is lacking. Therefore, considering the increasing consumer interest in this fruit, the aim of this work was to evaluate the potential of FT‐IR spectroscopy, as quantitative analytical technique for the evaluation of TSS, ascorbic acid, acidity, phenol, anthocyanin, browning, and pH. In this study, PCA was used to extract features, which are given as inputs to predictive models like the ν‐SVR, PLS, and ANN. Finally, the ability of each model to predict the internal parameters of mulberry juice is examined.

## 2 MATERIALS AND METHODS

### 2.1 Sample preparation

**FIGURE 1:**
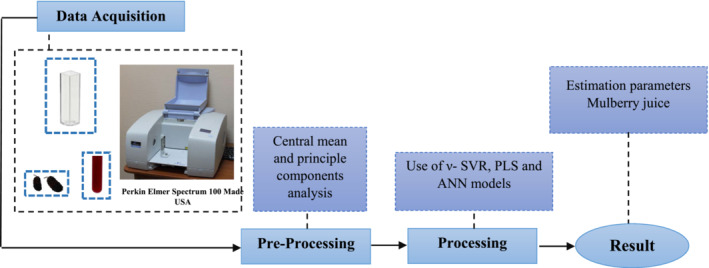
Flowchart of the approach to estimate quality parameters with ν‐support vector regression algorithm, PLS, and ANN

Mulberry fruits (*Morus nigra* L.) were manually harvested from mature trees of Agricultural Sciences and Natural Resources University of Khuzestan (Mollasani, 31°N, 48°E, and 35 Km northeast of Ahvaz, Iran). After harvesting, fruits were immediately transported to the laboratory. Fresh fruits were squeezed in by a home juicer. The pulp–juice mixture of each fruit was filtered through cotton pads and then centrifuged at 10,000 *g* for 10 min to remove the pulp. After that, they were stored at −4°C temperature until analysis. The total sample number was 100 and they were processed as shown in Figure 1.

### 2.2 Analysis of composition

Total soluble solids (TSS) were determined by a hand refractometer device (model: MT03 Japan) and expressed as °Brix. The anthocyanin content and browning were estimated following the procedure of Holcroft et al. (1998). The ascorbic acid of the juice was measured by titration with copper sulfate and potassium iodide based on Barakat et al.'s (1973) procedure. Titratable acidity was measured according to the AOAC method. To determine the total phenol content of juice, the Waterhouse method was used for the determination of the pH of the fruit extract using a pH meter (Portable Model P‐755, Japan) (AL Waterhouse, 2002).

### 2.3 FTIR spectroscopy

The measurements were performed using a spectrometer (Perkin Elmer Spectrum 100 model and US‐made US) at room temperature (20–25°C). The FT‐NIR spectra of mulberry juice samples were scanned over the range 1000–8333 nm with a resolution of 2 nm.

### 2.4 Principal component analysis

Principal component analysis (PCA) is a statistical procedure that uses an orthogonal transformation to convert a set of observations of possibly correlated variables into a set of values of linearly uncorrelated variables called principal components. The number of distinct principal components is equal to the smaller number of original variables or the number of observations minus 1. This transformation is defined in such a way that the first principal component has the largest possible variance, and each succeeding component in turn has the highest variance possible under the constraint that it is orthogonal to the preceding components. The resulting vectors are an uncorrelated orthogonal basis set. PCA is sensitive to the relative scaling of the original variables (Mat Nawi et al., 2013).

### 2.5 Partial least squares (PLS) analysis

Partial least square, which is an efficient statistical regression technique, was introduced almost 30 years ago to overcome collinearity (Chan et al., 2022). As a multivariate data analysis method, PLS has been widely employed in FT‐IR spectroscopy analysis. PLS analysis can be performed to create the regression model leading to the content prediction of chemical components. In this method, simultaneously, the matrix of independent (X) and dependent (Y) variables are considered. After preprocessing of the spectra, PLS models were developed related to the FT‐IR spectra and destructive values (i.e., the TSS, ascorbic acid, acidity, anthocyanin, browning, and pH) in each mulberry juice sample. In order to avoid overfitting the models, the calibration models were formulated with cross‐validation (k‐fold) according to the predictive residual error sum of squares (PRESS) statistic (Liu et al., 2010). The accuracy of FT‐IR models for fruit quality prediction is usually described by the value of the r, the root means square error of validation (RMSE) (Equation 1). A good model should have a lower RMSE and higher correlation coefficient (r or R) or coefficient of determination (*r* ^2^ or *R* ^2^) (Poon et al., 2012)

(1) $$\text{RMSE}=\sqrt{\frac{1}{n}\sum_{i=1}^{n}{\left(y_{\text{pred}}-y_{\mathrm{act}}\right)}^{2}}$$

where *n* is the number of spectra, *y*_act_ is the actual value, and *y*_pred_ is the predicted value of the fruit juice attribute.

### 2.6 Artificial neural network (ANN)

In this study, an anchor network structure with a hidden layer was used. In order to communicate between the input layer with the hidden layer and the hidden layer with the output, the sigmoid and linear function were used, respectively (Xu et al., 2022). The Levenberg–Marguardt learning principle was utilized to train the network (Steck et al., 2022). For training, data were randomly divided into two parts, so that two‐thirds (*N* = %70) and one‐third (*N* = %30) of the data were selected for training and testing the network, respectively. The input of the model was the selected principal components of the spectral data, and the outputs were TSS, ascorbic acid, acidity, phenol, anthocyanin, browning, and pH. In this study, the number of neurons in hidden layers was obtained using trial‐and‐error method. The model's performance was calculated by the coefficient of determination and mean square error validation (RMSE).

### 2.7 Support vector regression (SVR)

Models of support vector machines are divided into two main groups: (a) a support vector machine and (b) a support vector regression model. Support vector machine models are used to solve the classification problems of data into different classes, and the support vector regression model is used to solve prediction problems. Vipink used a new error function to construct the regression vector of the support machine, which is called the ε‐insensitive error function and is defined as (Awad & Khanna, 2015):

(2) $$L(y,f\left(x,\alpha \right)={\left|y-f\left(x,\alpha \right)\right|}_{\varepsilon }=\left\{\begin{array}{l} 0\;\text{if}\;\left|y-f\left(x,\alpha \right)\right|\leq \varepsilon \\ \left|y-f\left(x,\alpha \right)\right|-\varepsilon \;\text{otherwise} \end{array}\right\}$$

**FIGURE 2:**
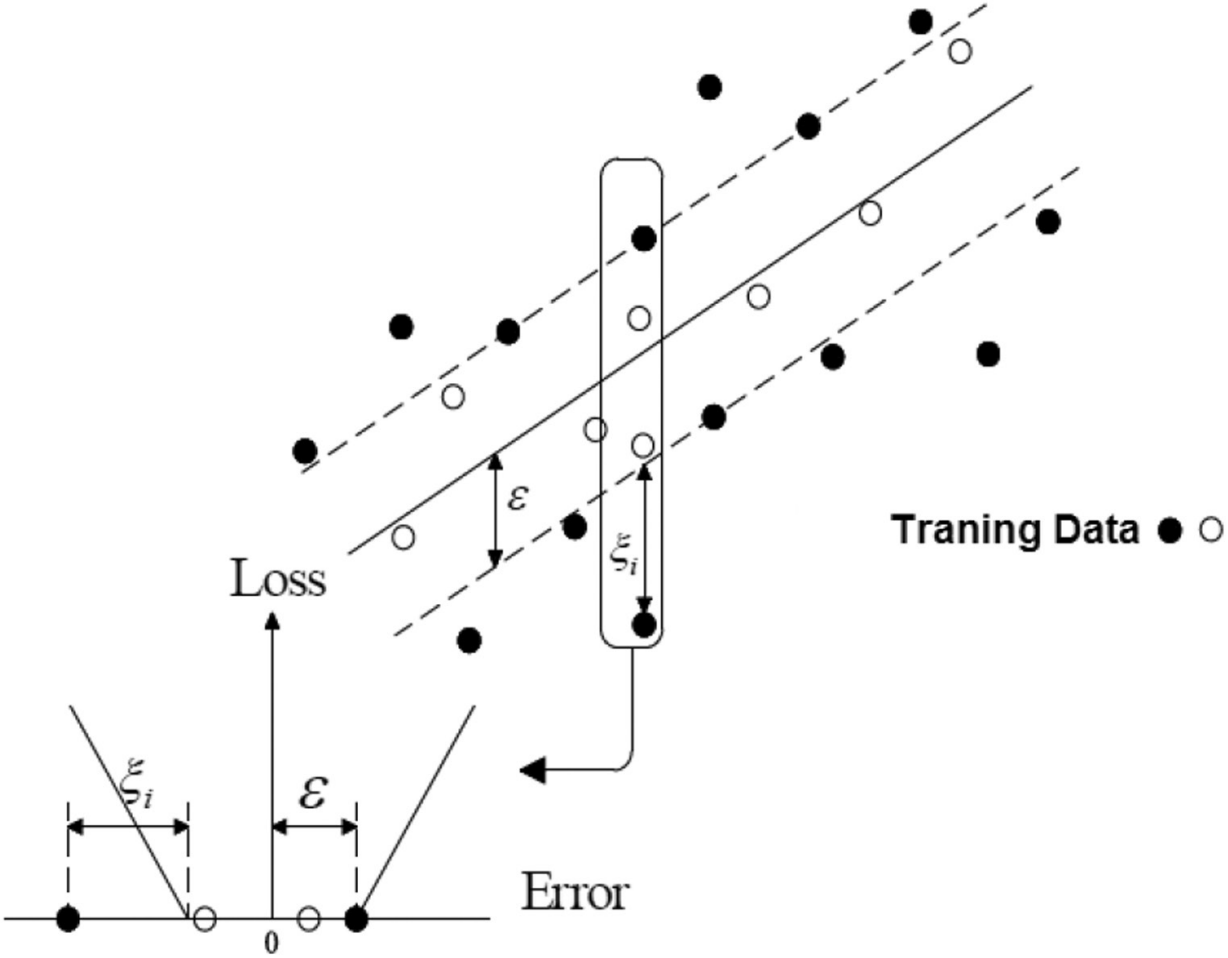
Error function ε‐insensitive

According to Equation 2, it can be seen that errors with values less than ε are not considered (Figure 2). In other words, in this function, errors in the lower limit ɛ are not penalized. It is called the pipe ε, and in multidimensional cases, the ε‐insensitive region has a shape such as a log, or in general, this space lies between two parallel cloud planes. For the development of the algorithm, a linear function must first be evaluated. All of the linear functions are as follows:

(3) $$f\left(x\right)=\left\langle w,x\right\rangle +b,\begin{array}{ccc} w,x\in X, & b\in R & \end{array}$$

In Equation 3, <..,..> specifies the inner multiplication of two vectors (*w* is the weight vector and *x* is the input space). The purpose of the learning process is to find the *f* function with the least error based on independent data with uniform distribution $\left(x_{1},y_{1}\right),\ldots ,\left(x_{m},y_{m}\right)\subseteq X,Y$, called the ε‐SVR algorithm. To this end, the *R*_reg_ generalized error function was minimized based on the ε‐insensitive error function (Equation 4). *R*_reg_ can be rewritten on an open form $R_{\mathrm{emp}}^{\varepsilon }$ (Awad & Khanna, 2015).

(4) $$R_{\mathrm{emp}}^{\varepsilon }\left[f\right]=\frac{1}{m}\sum_{i=1}^{m}{\left|y_{i}-f\left(x_{i}\right)\right|}_{\varepsilon }$$

$$R_{\mathrm{reg}}^{\varepsilon }\left[f\right]=\frac{1}{2}{\left\|w\right\|}^{2}+C.R_{\mathrm{emp}}^{\varepsilon }\left[f\right]$$

In this regard, $R_{\mathrm{emp}}^{\varepsilon }$ calculates the training error in the form of the ε‐insensitive error function, and C is a constant coefficient which is somehow a value of || w ||^2^ depending on the complexity of the function. The minimization of Equation (4) represents the basic idea of the theory of statistical learning that it expresses. To achieve the lowest real error, controlling the complexity of the model as well as the boundary data error is possible if and only if the estimation function has a limited capacity. This can be defined by introducing ineffective variables $\xi_{i},\xi_{i}^{*}\begin{array}{c} i=1,..,n \end{array}$.

$$\min\frac{1}{2}{\left\|w\right\|}^{2}+C\sum_{i=1}^{n}\left(\xi_{i}+\xi_{i}^{*}\right)$$

(5) $$s.t.\left\{\begin{array}{l} y_{i}-f\left(x_{i},w\right)\leq \varepsilon +\xi_{i}^{*}\\ f\left(x_{i},w\right)-y_{i}\leq \varepsilon +\xi_{i} \end{array}\right\}$$

$$\xi_{i},\xi_{i}^{*}\geq 0,i=1,\ldots ,n$$

The problem of minimizing the pseudoerror after transforming it into a minimization function and constructing the desired Lagrange expression is converted into a convex boundary optimization problem, which by moving its partial derivatives relative to decision variables is portable. Deviation from training samples outside the ε‐insensitive area is measured. In order to optimize the machine vector machine, the following formula was used (Equation 6):

(6) $$f\left(x\right)=\sum_{i=1}^{\mathrm{nsv}}\left(\alpha_{i}-\alpha_{i}^{*}\right)K\left(x_{i},x\right)\begin{array}{cc} \begin{array}{cc} & s.t.0\leq \alpha_{i}^{*}\leq C, \end{array} & 0\leq \alpha_{i}\leq C \end{array}$$

Where ɛ supports the vector number and ɣ the kernel function (Equation 7):

(7) $$K\left(x,x_{i}\right)=\sum_{j=1}^{m}\varphi_{j}\left(x\right)\varphi_{j}\left(x_{i}\right)$$

And $\alpha_{i}^{*}$ Lagrange is multivariate.

After solving the optimization problem, the values w and *f* are obtained as follows (Equation 8):

(8) $$w=\sum_{i=1}^{m}\left(\alpha_{i}^{*}-\alpha_{i}\right)x_{i}$$

$$f\left(x\right)=\sum_{i=1}^{m}\left(\alpha_{i}^{*}-\alpha_{i}\right)\left\langle x_{i},x\right\rangle +b$$

Before a linear split, the data are transmitted to a much larger space by the function *φ* for the machine to predict the complexity of the data. To solve a very high‐dimensional problem, the Lagrange duality theorem is used to transform the desired minimization problem into its duality form, in which instead of the complex function *φ* that goes up to a dimensional space, a simpler function called the core function (Kernel) which is a function of the function *φ*. Various kernel functions, including linear, radial, polynomial, and sigmoid nuclei, can be used. Therefore, it is sufficient to use the input values in the nonlinear problems of the kernel. According to the theory explained, the accuracy of determining the parameters of the smoothing factor C, the amount of the parameters of the term in the kernel function, has a significant effect on reducing the error of the problem. Statistical analysis was performed using software SPSS as well as all analyzes by MATLAB software.

## 3 RESULTS AND DISCUSSION

### 3.1 Changes in qualitative parameters studied during fruit maturity

According to the statistical analysis of destructive parameters (Table 1), the amount of TSS, total anthocyanin, and browning increased as ripeness changed from stages 1 to 4, but the amount of phenol, acidity, and ascorbic acid decreased significantly (*p* < .05) in the maturity process. The results of this study are in agreement with the findings of Zheng et al. (2011) for oatmeal and Özgen et al. (2009) for mulberry fruit. During grape development, TA usually decreases as TSS increases (Daniels et al., 2019). However, the pH changes were fluctuating during the treatment period and no specific pattern was observed. According to the results of Table 1, it can be seen that different stages of maturity have a significant effect on the mulberry traits.

**TABLE 1:** Comparison of mean values for destructive parameters (TSS, ascorbic acid, acidity, phenol, total anthocyanin, browning, and pH)

Treatment	Mean square						
	TSS	Ascorbic acid (mg‐100)	Acidity (%)	Phenol (mgr/100 gr)	Anthocyanin (AU/ml)	Browning	pH
Stage 1	5.05^c^	47.17^a^	0.125^a^	419.67^c^	1.3113^c^	2.12^d^	3.014^b^
Stage 2	5.82^b^	42.46^b^	0.0205^c^	468.01^c^	0.6080^c^	3.04^c^	2.97^c^
Stage 3	6.085^b^	22.92^c^	0.055^b^	920.98^a^	12.43^b^	7.95^b^	3.07^a^
Stage 4	10.69^a^	21.35^c^	0.015^d^	684^b^	21.46^a^	11.051^a^	2.175^d^

*Note*: Means in each column followed by a similar letter (s) are not significantly different at 5% probability level using Duncan's multiple‐range test.

### 3.2 Spectrum obtained during fruit maturing

**FIGURE 3:**
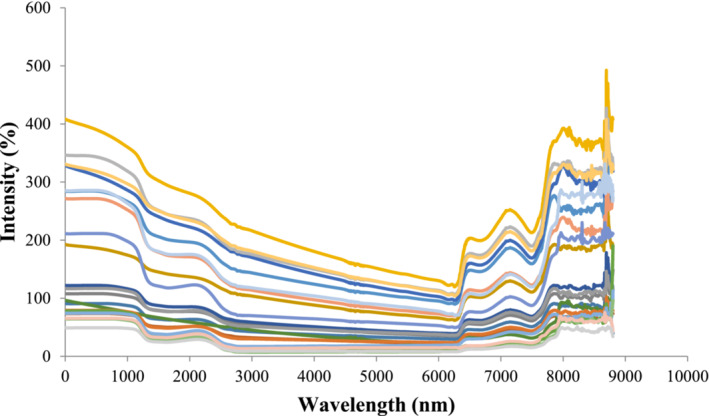
Spectral dataset after central mean preprocessing

The spectra were corrected and noises were removed using the central meaning preprocessing method (Figure 3).

### 3.3 Determine the number of principal components using cross‐validation

**FIGURE 4:**
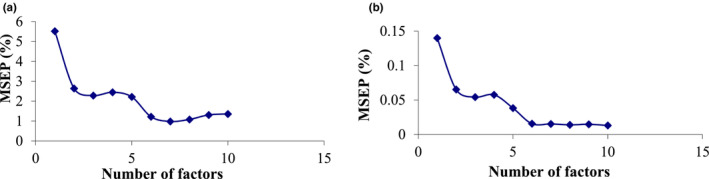
The number of factors and MSEP for (a) TSS and (b) pH

Considering the importance of determining the number of principal components, the most effective number was extracted. In this study, k‐fold cross‐validation as well as prediction of error estimation were used to determine the most efficient number of principal components. Furthermore, the k‐value was determined to be 10 with respect to the lowest mean square error estimate using trial‐and‐error method. For example, Figure 4 shows the plots of prediction error values for TSS and pH parameters as a function of the number of principal components. As outlined in this Figure 3, the first six principal components had the least prediction error, and therefore, they were given as inputs to the models. It is worth noting that more than 95% of the total variance is covered by six PCs in the analysis. Clearly, the PCR method provides a more accurate response with more number of components; however, the number of more and less components, in practice, leads to overfitting and underfitting, respectively. Therefore, in order to increase the accuracy of the model and prevent overfitting and underfitting, six PCs were selected.

### 3.4 ν‐Support vector regression model

**FIGURE 5:**
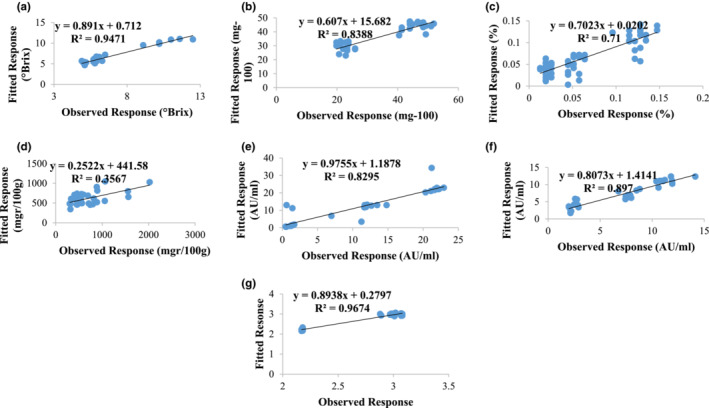
Scatter plot between observed values and predicted FT‐IR values of SVR modeling, (a) TSS, (b) ascorbic acid, acidity, (d) phenol, (e) anthocyanin, (f) browning, and (g) pH in mulberry juice

For training the model, two‐thirds (*N* = %70) of the data were used and one‐third (*N* = %30) were used for testing. The ability of the model's performance was evaluated by correlation coefficient and mean error of validation. The first six selected PCs were entered as inputs to the SVR model. According to Figure 5, the SVR training models of TSS, ascorbic acid, acidity, phenol, anthocyanin, browning, and pH parameters have a high coefficient of determination of 0.94, 0.84, 0.71, 0.35, 0.93, 0.89, and 0.96; and training root mean error of 0.014, 0.29, 0.0004, 0.19, 5.85, 0.005, 0.062, and 0.005 respectively. The *R* ^2^ value of soluble solids content acquired, in this research, is the same as obtained by Lafuente et al. (2019) for nondestructive determination of soluble solids content in Prunus avium (*R* ^2^_cv_ = .98) using Vis/NIR equipment.

### 3.5 Partial least square prediction model

**FIGURE 6:**
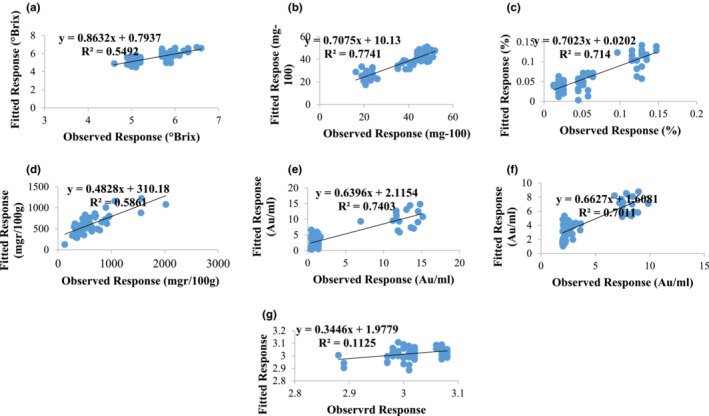
Scatter plot between observed values and predicted FT‐IR values of PLSR modeling, (a) TSS, (b) ascorbic acid, (c) acidity, (d) phenol, (e) anthocyanin, (f) browning, and (g) pH in mulberry juice

The coefficient of determination of PLS training model for the parameters of TSS, ascorbic acid, acidity, phenol, total anthocyanin, browning process, and pH were 0.54, 0.77, 0.71, 0.58, 0.74, 0.70, and 0.11, respectively; and the MSE were 0.005, 1.41, 0.001, 21.63, 0.55, 0.32, and 0.003, respectively (Figure 6a–h). According to the results, this model was able to predict the ascorbic acid, acidity, total anthocyanin, and browning process parameters well.

The *R*‐value of total anthocyanin obtained in this research from spectral data was moderately better than those obtained by Larraín et al. (2008) with *R* between .79 and .83 in different grape cultivars using spectra ranging from 640 to 1100 nm. Moreover, in a study by Daniels et al. (2019) using Fourier transform near‐infrared (FT‐NIR) spectroscopy on grapes' internal parameters, the PLSR model was able to predict TSS, titratable acidity, and pH with the prediction correlation coefficient of 0.71, 0.33, and 0.28, respectively. The results of this research were also better than those of Baiano et al. (2012) and González‐Caballero et al. (2010), who obtained *R* ^2^ of .80 and .51 for the prediction of the pH values in grapes and wine grapes, respectively.

### 3.6 ANN prediction model

**FIGURE 7:**
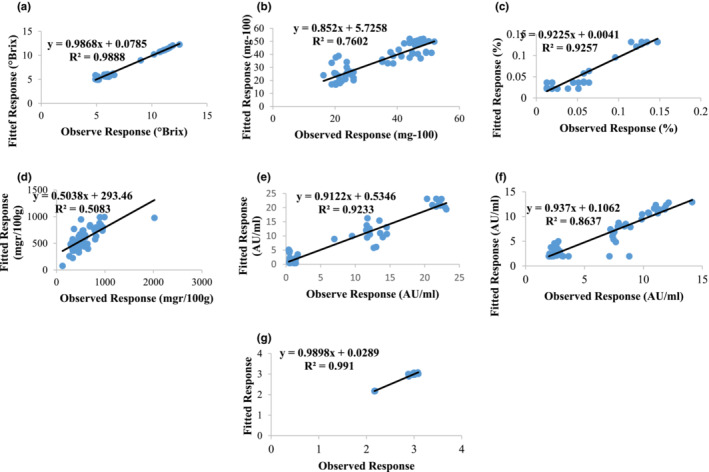
Scatter plot between observed values and predicted FT‐IR values of ANN modeling, (a) TSS, (b) ascorbic acid, (c) acidity, (d) phenol, (e) anthocyanin, (f) browning, and (g) pH in mulberry juice

In order to train ANN, the first six PCs were given as inputs into the ANN prediction model. The number of neurons in the hidden layer were 12, 19, 6, 6, 4, 6, and 13, respectively, for the biochemical parameters of total soluble solids, ascorbic acid, acidity, phenol, total anthocyanin, browning process, and pH. The number of neurons in the hidden layer was determined by trial‐and‐error method. Plots of predicted chemical parameters as obtained from the ANN training stage versus observed values are shown in Figure 7a–h. The means square error for the neural network for the chemical characteristic of total soluble solids, ascorbic acid, acidity, phenol, total anthocyanin, browning process, and pH were 0.003, 0.062, 0.0003, 0.50, 1.02, 0.02, and 0.0002, respectively, and the correlation coefficients of 0.98, 0.76, 0.92, 0.50, 0.92, 0.86, and 0.99, respectively. He et al. (2013) employed hyper spectral images in the 900–1700 nm wavelength range for the prediction of SSC in jujubes. According to the result, the BP‐ANN trained with five characteristic wavelengths which were identified by PCA yielded an *R* of .90 and RMSEP of 1.98.

### 3.7 Comparison of ν‐SVR, PLSR, and ANN models in order to predict the quality parameters of mulberry juice

The ability of SVR, PLSR, and ANN models for the prediction of TSS, ascorbic acid, acidity, phenol, anthocyanin, browning, and pH various maturity stages was compared with each other (Table 2). SVR model predicted ascorbic acid (*R* ^2^ = .84, RMSE = 0.29), acidity (*R* ^2^ = .71, RMSE = 0.0004), phenol (*R* ^2^ = .35, RMSE = 0.19), total anthocyanin (*R* ^2^ = .93, RMSE = 5.85), and browning (*R* ^2^ = .89, RMSE = 0.062) parameters with acceptable performance; however, the ANN model was able to predict parameters TSS (*R* ^2^ = .98, RMSE = 0.003) and pH (*R* ^2^ = .99, RMSE = 0.0009) better than SVR and PLSR models. Based on the results, the PLSR model had lower performance in predicting the parameters than the other two models (SVR and ANN). Thus, model SVR estimated the parameters TSS, ascorbic acid, acidity, anthocyanin, and browning with better performance than models ANN and PLSR. But the ANN model predicted the pH parameter with a high percentage of *R* ^2^ = .97 and the lowest RMSE = 0.009 value.

**TABLE 2:** Results of SVR, PLS, and ANN models

Model	Test	Parameters						
		TSS	Ascorbic acid	Acidity	Phenol	Anthocyanin	Browning	pH
SVR	*R* ^2^	.96	.82	.90	.41	.92	.84	.92
	MSE	0.53	3.4	0.00008	2.77	0.0049	0.03	0.013
PLSR	*R* ^2^	.91	−.97	.56	.60	.88	.69	.95
	MSE	0.033	0.12	5.6 e‐05	37.82	0.59	0.18	0.009
ANN	*R* ^2^	.87	.75	.89	.24	.89	.83	.97
	MSE	0.025	0.51	0.0002	0.02	0.04	0.01	0.0002

This result falls in line with the research of Sanaeifar et al. (2016) with better results for the prediction of TSS and pH and acidity during the storage of bananas. Also, in a research by Xiaobo et al. (2007), the comparison of the SVR to determine the SSC in apple with PLSR and ANN was conducted. According to the results of the three models, the SVR model was selected as the best model for determining the SSC parameter with a correlation coefficient of 0.95.

## 4 CONCLUSION

The primary objective of this work was to develop predictive models for the measurement of quality parameters of mulberry fruit juice at different maturity stages. To do so, FT‐IR spectroscopy in combination with PLS, SVR, and ANN was conducted to predict the chemical parameters of mulberry juice (TSS, ascorbic acid, acidity, phenol, anthocyanin, browning, and pH). According to the statistical analysis, the chemical parameters TSS, ascorbic acid, anthocyanin, and browning increased, and the parameters of acidity and pH decreased, significantly. The results showed that the performance of the SVR model was better in prediction parameters of ascorbic acid, acidity, phenol, total anthocyanin, and browning than in PLS and ANN models. However, ANN predicted the parameters TSS and pH better than the SVR and PLS models. Furthermore, in concordance with the results obtained for calibration and prediction parameters, the three models can be used for the quantification of parameters of mulberry juice.

## CONFLICT OF INTEREST

The authors declare that they have no conflict of interest regarding the publication of this manuscript.

## Data Availability

The datasets generated during and/or analyzed during the current study are available from the corresponding author upon reasonable request.
